# Propofol exposure during late stages of pregnancy impairs learning and memory in rat offspring *via* the BDNF‐TrkB signalling pathway

**DOI:** 10.1111/jcmm.12884

**Published:** 2016-06-14

**Authors:** Liang Zhong, Foquan Luo, Weilu Zhao, Yunlin Feng, Liuqin Wu, Jiamei Lin, Tianyin Liu, Shengqiang Wang, Xuexue You, Wei Zhang

**Affiliations:** ^1^ Department of AnesthesiologyThe First Affiliated HospitalNanchang UniversityNancahangChina

**Keywords:** propofol, late pregnancy, offspring, learning and memory, BDNF‐TrkB signal pathway, 7,8‐dihydroxyflavone, synaptophysin

## Abstract

The brain‐derived neurotrophic factor (BDNF)‐tyrosine kinase B (TrkB) (BDNF‐TrkB) signalling pathway plays a crucial role in regulating learning and memory. Synaptophysin provides the structural basis for synaptic plasticity and depends on BDNF processing and subsequent TrkB signalling. Our previous studies demonstrated that maternal exposure to propofol during late stages of pregnancy impaired learning and memory in rat offspring. The purpose of this study is to investigate whether the BDNF‐TrkB signalling pathway is involved in propofol‐induced learning and memory impairments. Propofol was intravenously infused into pregnant rats for 4 hrs on gestational day 18 (E18). Thirty days after birth, learning and memory of offspring was assessed by the Morris water maze (MWM) test. After the MWM test, BDNF and TrkB transcript and protein levels were measured in rat offspring hippocampus tissues using real‐time PCR (RT‐PCR) and immunohistochemistry (IHC), respectively. The levels of phosphorylated‐TrkB (phospho‐TrkB) and synaptophysin were measured by western blot. It was discovered that maternal exposure to propofol on day E18 impaired spatial learning and memory of rat offspring, decreased mRNA and protein levels of BDNF and TrkB, and decreased the levels of both phospho‐TrkB and synaptophysin in the hippocampus. Furthermore, the TrkB agonist 7,8‐dihydroxyflavone (7,8‐DHF) reversed all of the observed changes. Treatment with 7,8‐DHF had no significant effects on the offspring that were not exposed to propofol. The results herein indicate that maternal exposure to propofol during the late stages of pregnancy impairs spatial learning and memory of offspring by disturbing the BDNF‐TrkB signalling pathway. The TrkB agonist 7,8‐DHF might be a potential therapy for learning and memory impairments induced by maternal propofol exposure.

## Introduction

It was traditionally thought that the effects of general anaesthetics were reversible. However, as a result of the *Science* publication by Ikonomidou *et al*. 1 showing *N*‐methyl‐d‐aspartic acid (NMDA) antagonist neurotoxicity in developing neurons, the occurrence of long‐term damage to the central nervous system (CNS) has gained much attention and has become a new research focus in the anaesthesiology and neuroscience fields. Increasing evidence indicates that most general anaesthetics can induce neuronal apoptosis and long‐term neurobehavioural impairments in developing brains and in young animals 2, 3, 4, 5. Recent clinical evidence suggests that general anaesthesia may impair long‐term neurodevelopmental outcome in preterm babies 6.

Propofol is one of the most commonly used intravenous general anaesthetics and is used for paediatric anaesthesia. Increasing clinical evidence has shown that patients can exhibit symptoms of cognitive dysfunction several weeks or even several months after exposure to propofol anaesthesia 7, 8. Animal experiments have also shown that propofol anaesthesia can induce neuronal apoptosis in the brain as well as permanent cognitive deficits 9. Exposure of newborn or young rodents to propofol can also result in learning and memory impairments after adulthood in a dose‐dependent manner 10, 11, which is associated with neuronal damage in the brain 12.

The exact mechanism for the long‐term effects of propofol on cognitive function still needs further clarification. Synaptic morphology and function is the basis of learning and memory. There are two different forms of synaptic plasticity: long‐term potentiation (LTP) and long‐term depression (LTD). Balancing the expression and biological function of LTP and LTD is critical for the correct formation and maintenance of learning and memory. Formation of LTP requires the involvement of brain‐derived neurotrophic factor (BDNF), which regulates neuron development in the CNS and is closely related to learning and memory 13, 14, 15, 16. Mizuno *et al*. 17 have demonstrated that the acquisition of learning and memory is accompanied by an increase in BDNF mRNA expression in the rat hippocampus. Compared with the control group, the phosphorylation levels of the BDNF tyrosine kinase B (TrkB) receptor in the hippocampus were selectively increased in parallel with the acquisition of learning and memory. Rats with BDNF defects showed impairments both in hippocampus LTP and hippocampus‐dependent learning and memory 18. Brain‐derived neurotrophic factor is involved in the regulation of learning and memory by binding to the TrkB receptor 19. It is known that the BDNF‐TrkB signalling pathway is involved in the plasticity of learning and memory 20. Binding of BDNF to the TrkB receptor leads to autophosphorylation of the receptor and subsequent activation of cytoplasmic signalling pathways, including mitogen‐activated protein kinase (MAPK), thus facilitating the synthesis and release of neurotransmitters that contribute to learning and memory. When BDNF binds to TrkB in the postsynaptic membrane, NMDA and alpha‐amino‐3‐hydroxy‐5‐methyl‐4‐isoxazolepropionic acid (AMPA) receptors are subsequently activated, which increases the phosphorylation level of NMDA receptor subunits NR1 and NR2B, promotes the expression of AMPA receptor subunits GluR1 and GluR2/3 (which enhances hippocampal synaptic transmission and efficacy), and finally promotes learning and memory 21. It is known that synaptophysin, a synaptic protein marker, is dependent on BDNF processing and subsequent TrkB signalling pathways 22 and plays an important role in learning and memory. Propofol has been shown to suppress LTP by inhibiting the expression of synaptic plasticity‐related proteins in the hippocampus CA1 region in neonatal mice 23.

Pregnancy is the key period for prenatal brain development, during which, the foetus is vulnerable to external noxious stimuli 24. The key period for neuron proliferation and synaptic growth varies from species to species. For humans, the neuron proliferation and growth period is mainly from the last trimester to 2 years after birth; however, in rats, the essential period ranges from 2 days before delivery to 2 weeks after delivery 25. Therefore, later stages of pregnancy are essential for neuron structure and function development, and the CNS is very sensitive to internal and external noxious stimuli 26. Approximately 2% of pregnant women undergo nonobstetric surgery due to various clinical conditions 27, 28, 29. There are approximately 75,000 nonobstetric surgeries in the United States and 76,000 in Europe 30. Most of these procedures must be completed under general anaesthesia 31. Propofol is the most widely used intravenous general anaesthetic and has been used in pregnant woman for sedation 32, 33, cesarean delivery 34, 35, 36 and/or nonobstetric surgery 37, 38. It is well‐known that propofol can easily pass through the placental barrier 39. Therefore, the effects of prenatal general anaesthetic exposure on learning and memory must be understood. Clinical data have indicated that nonobstetric surgeries during pregnancy are safe for the mother and foetus 40, 41, 42, 43, 44, 45, 46, 47, 48. However, most studies have been focused on physical development; therefore, little is known about the long‐term outcome and effects on learning and memory in the developing foetus. Most of the published studies about the long‐term effects of propofol on the CNS have mainly focused on individuals who directly received propofol. Propofol causes less cardiac injury than isoflurane 49, and compared to isoflurane in preterm lambs suffering from severe asphyxia, propofol has also been shown to improve foetal electroencephalogram (EEG) test results by down‐regulating NMDA receptors and inhibiting the mitochondrial apoptotic pathway 50. However, little is known about the effects of maternal exposure to propofol during pregnancy on learning and memory in offspring. Our previous studies have shown that maternal exposure to propofol for 2 hrs during early gestation had no significant effects on learning and memory or the expression of genes, including c‐fos and c‐jun, in offspring; however, 4 hrs of propofol exposure caused significant learning and memory impairments 51. It has been reported that the permanent learning and memory impairments that are induced in offspring following maternal propofol exposure may correlate with increased levels of cleaved caspsase‐3, reduced neurons and decreased synaptophysin 1 levels in the hippocampus 52; however, the role of the BDNF‐TrkB signalling pathway is not yet known. In this study, we investigate the effects of maternal propofol exposure at day E18 on learning and memory and the expression of BDNF, TrkB and synaptophysin in rat offspring. Additionally, we investigate whether the TrkB receptor agonist 7,8‐DHF can modulate these effects.

## Materials and methods

### Chemicals

The following main reagents were used in this study: propofol (Diprivan, AstraZeneca UK limited, Italy: jc393, 20 ml: 200 mg); 20% intralipid (2B6061; Baxter, Deerfield, IL, USA); 7,8‐Dihydroxyflavone (50 mg; Abcam, Cambridge, MA, USA); dimethyl sulphoxide (DMSO, 100 ml; Takara, Japan); TRIzol total RNA extraction kit (100 ml; Takara, Ohtsu, Shiga, Japan); RNAstore (100 ml; Takara); diethylpyrocarbonate (DEPC) (100 ml; Takara); MD101‐02 DNA Maker I (2 ml; Takara); Prime ScriptTM RT Reagent Kit with gDNA Eraser (Perfect Real Time) (Takara Code: RR047A for 100 * 20 μl reactions, Takara); SYBR Premix Ex TaqTM II (Tli RNaseH Plus) (Takara Code: RR820A for 200 * 50 μl reactions, Takara); BDNF, TrkB and β‐actin RT‐PCR primers (Sigma‐Aldrich, MA, USA); and DAB Colour Developing Reagent Kit for immunohistochemistry (IHC; DAKO, Carpinteria, CA, USA). Brain‐derived neurotrophic factor primary antibody (50 ug), rabbit polyclonal anti‐TrkB antibody (ab18987), rabbit polyclonal anti‐TrkB (phospho Y515) antibody (ab109684), rabbit polyclonal anti‐synaptophysin antibody (ab23754) and rabbit polyclonal anti‐β‐Actin antibody (ab8227) were purchased from Abcam Company.

### Animals

**Figure 1:**
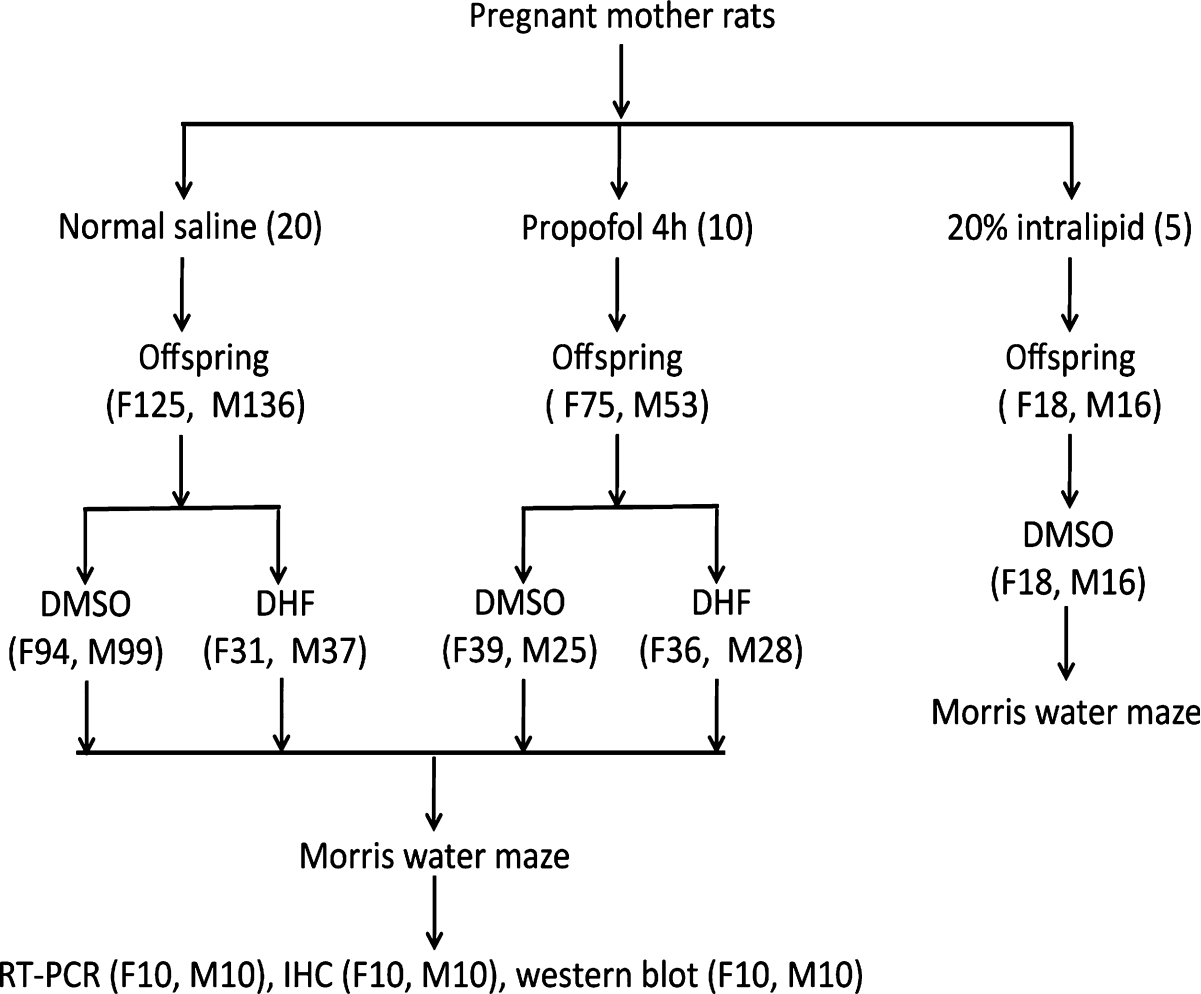
The flow chart of the experimental protocols and distribution of offspring rats among different studies. The number in bracket stands the number of animals. F: female; M: male; DMSO: dimethyl sulphoxide; DHF: 7,8‐dihydroxyflavone; RT‐PCR: real time‐polymerase chain reaction; IHC: immunohistochemisry.

Sprague‐Dawley (SD) rats were supplied by the animal science research department of the Jiangxi Traditional Chinese Medicine College (JZDWNO: 2011‐0030). Animals were maintained under the standard conditions of temperature between 22°C and 25°C, humidity at 55 ± 5%, and daily 12 hrs light/12 hrs dark cycle (with lights on at 7:00–19:00). Animals received food and water *ad libitum*. To exclude the possible contribution of genetic factors on the offspring, parental rats were assessed for learning and memory function using the Morris water maze (MWM) to ensure that there were no significant cognitive differences. Based on the learning and memory results, parental rats were then assigned to propofol (*n* = 10), control (*n* = 20) or intralipid (*n* = 5) treatment groups (Fig. 1). Female and male rats were housed together to allow for mating (2 female rats and 1 male rat per cage).

### Propofol exposure

On day E18, 20 mg/kg propofol were injected into gestating rats in the propofol exposure group *via* the caudal vein and was followed by 20 mg/kg/hr of continuous infusion for 4 hrs. Equal volumes of saline were given to rats in the control group, while 20% intralipid was given to the intralipid group. The propofol infusion time was selected based on the following information: (*i*) It is proven that neuronal damage is highest when general anaesthetic exposure time is prolonged to between 6 and 8 hrs, but a 2 hrs exposure time has no significant effects; (*ii*) Most surgeries require 2–4 hrs of anaesthesia; and (*iii*) Our previous studies have shown that maternal exposure to propofol (at the same dosage used in this study) for 2 hrs during gestation had no significant effects on learning and memory in offspring, but exposure for 4 hrs caused significant impairments without affecting the levels of arterial blood gases of pregnant rats 51.

Electrocardiograms, saturation of pulse oximetry (SpO_2_) and tail non‐invasive blood pressure were monitored during the time of propofol exposure. Tail arterial blood was collected for blood gas analysis after propofol infusion. If the cumulative time of SpO_2_ was <95% and/or the systolic blood pressure was <80% of baseline for more than 5 min., the maternal rat was removed from the study, and another rat was selected to supplement the sample size, thereby preventing toxicity from maternal ischaemia or hypoxia in rat offspring.

### Morris water maze test

Spatial memory and learning of rat offspring were tested by the MWM test at postnatal day 30 (P30). The trials began at 9:00 am in a MWM system that was filled with water at 24 ± 1°C. A platform was hidden 1 cm below the water surface in the second quadrant (*i.e*., the target quadrant) of the MWM system. Each rat was put into the pool and allowed to search for the platform one time per day for six consecutive days. The time for the rats to find the platform was recorded as the escape latency (which indicated learning ability). The animals were allowed to stay on the platform for 30 sec. if they were able to find the platform within 120 sec. However, if an animal did not find the platform within 120 sec., the animal was guided to the platform, allowed to remain there for 30 sec., and the escape latency was recorded as 120 sec. On the seventh day, the platform was removed. The rats were then allowed to perform the spatial probe test (a memory test) for 120 sec. The number of times that the rat swam across the site where the platform was hidden (number of platform‐crossing times) and the time spent in the target quadrant (target quadrant time) were recorded with a video connected to a computer. The mean value of the latencies, platform‐crossing times or target quadrant times of the offspring born to the same mother were calculated as the final results.

### 7,8‐DHF injection

**Figure 2:**
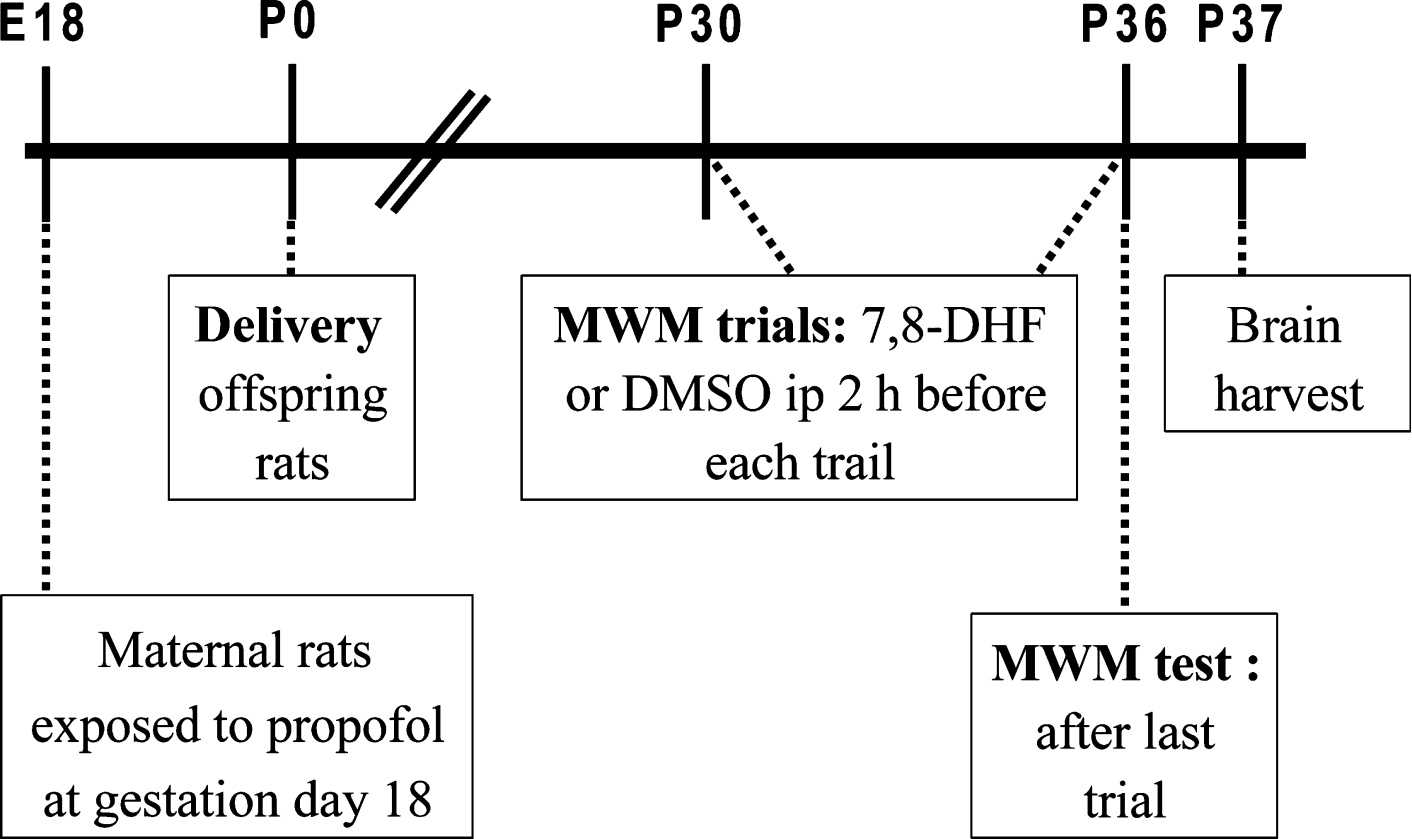
Schematic time‐line of Morris Water Maze tests paradigm. E18: pregnant rats at gestational day 18; P0: postnatal day 0; MWM: Morris water maze; 7,8‐DHF: 7,8‐dihydroxyflavone; DMSO: dimethyl sulfoxide.

On day P30, the rat offspring were randomly subdivided into the TrkB agonist group (PD and CD group, respectively) and vehicle group (P group and C group, respectively) (Figs 1 and 2). Two hours before each MWM trial, 5 mg/kg of 25.42 mg/ml of 7,8‐DHF in DMSO were given intraperitoneally to the offspring in the PD and CD groups. Equal volumes of DMSO were given to rats in the P and C groups. The dosage and the time point selection were based on knowledge that 7,8‐DHF can pass the blood‐brain barrier. Furthermore, a 5 mg/kg intraperitoneal injection administered 2 hrs before middle cerebral artery occlusion has been shown to reduce neuron death caused by cerebral ischaemia and also significantly reduces the infarction area by activating the TrkB receptor 53. Behavioural analyses have proven that intraperitoneal injection of 7,8‐DHF administered 2 hrs before noxious stimuli has been shown to prevent spatial memory impairments induced by immobilization stress 54.

### Brain harvest

The day after the MWM test, rats were anaesthetized and killed by cervical dislocation. Hippocampus tissues were harvested and stored in Eppendorf tubes that had been treated with 1% DEPC and were stored at −80°C (for RT‐PCR and western blot analyses) or immersed in 4% paraformaldehyde (for IHC experiments).

### Real‐time PCR

Total RNA was extracted with the TRIzol kit (Takara). cDNA synthesis was performed according to the manufacturer's instructions. Real‐time PCR was performed using the SYBR Green PCR Kit. The following primers were used: BDNF‐ forward primer (5′‐CCAACGAAGAAAACCATAG‐3′), reverse primer (5′‐AATACTGTCACACAGTCA‐3′); TrkB‐ forward primer (5′‐AGCATGAGCACATCGTCAAG‐3′), reverse primer (5′‐ATATGCAGCATCTGCGACTG‐3′); and β‐actin‐ forward primer (5′‐ACCACAGTCCAGCCATCAC‐3′), reverse primer (5′‐TCCACCACCCTGTTGCTGTA‐3′). The products of the PCR were analysed by the ABI 7500 system (Applied Biosystems, Waltham, MA, USA), and β‐actin was used as an endogenous control. The results were calculated by the ddCt method (2^−[(Ct of target gene)−(Ct of β‐actin)]^, where Ct is the threshold cycle for each transcript).

### Immunohistochemistry

The hippocampus tissues of the rat offspring were collected and embedded in paraffin, after immersion and fixation in 4% paraformaldehyde and were then cut into 5 μm thick sections. After deparaffinization, rehydration and antigen retrieval, the sections were incubated with the following primary antibodies at 37°C for 1 hr: rabbit polyclonal anti‐BDNF antibody (1:500) and rabbit polyclonal anti‐TrkB antibody (1:500). After incubation with the appropriate secondary antibody, the protein complex was labelled with DAB chromogenic reagent, coverslipped with permount and observed by an optical microscope. The HPIAS‐1000 high definition colour pathological image analysis system was used to analyse the images. Three sections were obtained from each rat. Three visual fields were randomly selected and observed for each tissue section. The mean grey value of the protein complex of the offspring born to the same mother was regarded as the protein expression level.

### Western blot

Total protein lysates were prepared by homogenizing hippocampal tissues (*n* = 10 in each group) in lysis buffer (Thermo Scientific, Rockford, IL, USA) containing a protease inhibitor cocktail (Sigma‐Aldrich) and phosphatase inhibitors (PhosSTOP Phosphatase Inhibitor Cocktail Tablets; Roche, Nutley, NJ, USA). Protein concentrations of samples were determined using the BCA protein assay (Bio‐Rad, Hemel Hempstead, Herts, UK). Twenty micrograms of each protein sample were analysed by western blot using the following primary antibodies: rabbit polyclonal antiphospho‐TrkB at 1:1000, rabbit monoclonal anti‐synaptophysin at 1:1000 and rabbit polyclonal anti‐β‐Actin at 1:5000. Images were scanned by an Image Master II scanner (GE Healthcare, Milwaukee, WI, USA) and were analysed using ImageQuant TL software v2003.03 (GE Healthcare). The signals for the protein bands of interest were normalized to those of β‐actin and were then expressed as fractions of the control samples from the same gel.

### Statistical analyses

Statistical Package for Social Sciences (SPSS) version 17.0 software (SPSS, Inc., Chicago, IL, USA) was used to analyse the data. Escape latency data were analysed by two‐way anova with repeated measurement, with prenatal treatment as a between‐litters independent factor and day as a repeated factor. When an initial anova showed effects of the factors and significant interactions among the factors, post hoc comparisons were conducted. Data for mRNA, protein and blood gases were analysed by one‐way anova. There was no missing data for any of the variables. The LSD *t*‐test was used to determine the difference between the control and propofol exposure conditions. The LSD *t*‐test was also used to assess the difference between the 7,8‐DHF and DMSO control conditions for the expression levels of BDNF, TrkB, phospho‐TrkB and synaptophysin. Values of *P* < 0.05 were considered statistically significant.

## Results

### Blood gas

To investigate whether 4 hrs of propofol exposure on day E18 can cause disturbances in maternal blood gases, caudal artery blood was collected from pregnant rats for blood‐gas analysis after propofol perfusion. It was discovered that there were no significant differences (*P* > 0.5) between the propofol exposure and control groups (Table 1), indicating that propofol infusion has no significant effects on blood gases in pregnant rats. Therefore, the results of the current study are likely caused directly by propofol instead of secondary effects of maternal propofol infusion.

**Table 1:** The comparison of blood gases between control and propofol maternal rat groups (*n* = 10, mean ± S.E.M)

Blood gases	Control maternal rats	Propofol maternal rats
PH	7.4 ± 0.0	7.4 ± 0.2
PO_2_ (mmHg)	108.0 ± 5.5	105.0 ± 4.8
PCO_2_ (mmHg)	51.3 ± 3.4	50.8 ± 4.4
HCO_3‐_ (mmol/l)	30.6 ± 0.8	28.4 ± 2.4
BE (B)	3.7 ± 1.4	3.5 ± 0.8
Ca^2+^ (mmol/l)	1.3 ± 0.2	1.3 ± 0.1
K^+^ (mmol/l)	4.5 ± 0.6	4.5 ± 0.7
Na^+^ (mmol/l)	135.5 ± 1.4	134.5 ± 1.1

### Physical features of the offspring

Propofol exposure in late stages of pregnancy had no effect on birth rate, offspring survival rate (the ratio of rat offspring that survived more than 30 days) or gender ratio. The litter number in the control and propofol groups was 276 and 139, respectively. The offspring survival rate of the control and propofol exposure groups was 94.6% (261/276) and 92.1% (128/139), respectively. The ratio of males to females in the control and propofol groups was 1.1 and 1.4, respectively. Maternal propofol exposure also had no obvious influence on offspring physical development. On day P30, the average weight of offspring in the control group (130.3 ± 10.9 g) was not significantly different from that in the propofol exposure group (132.7 ± 9.8 g). Dyskinesia was not observed in either of the two groups. These results indicate that propofol exposure on day E18 has no obvious effects on the offspring survival, sex ratio or basic physical development and suggests that the differences in learning and memory observed in the current study are induced by maternal propofol exposure instead of physical differences.

### Impaired learning and memory in offspring and the ameliorating effects of DHF treatment

**Figure 3:**
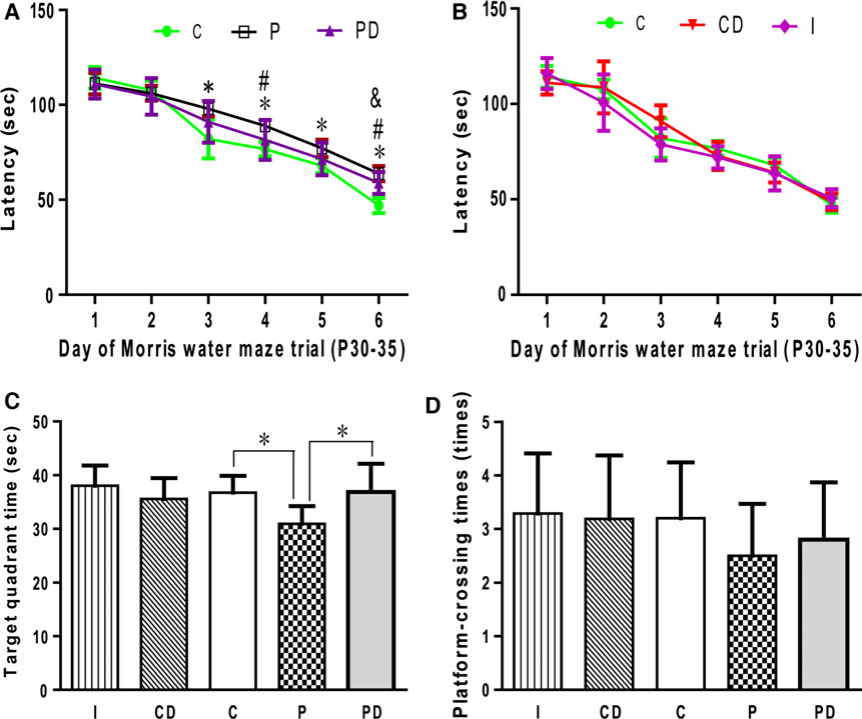
DHF reversed the impaired learning and memory. On P30, the learning and memory of offspring rats were tested by Morris water maze (MWM). Compared to the control group, the offspring in the propofol exposure group required additional time (escape latency) to find the hidden platform at the 3rd, 4th, 5th and 6th trials (**A**), but had shorter target quadrant travelling time (**C**) and shorter platform‐crossing times (**D**). Learning and memory in the 20% intralipid group (I group) was not significantly different from that in the control group (**B**,** C**, and **D**). 5 mg/kg of 7,8‐DHF was intraperitoneally injected in rat offspring 2 hrs before each MWM trial. The results showed that the offspring in the PD group had shorter escape latency than those in the P group at the 4th and 6th trials (**A**), while had more target quadrant time that P group (**C**). However, the escape latency in the PD group was still worse than that in the control group at the 6th trial (**A**). 7,8‐DHF had no significant effect on learning and memory in rat offspring that were not exposed to propofol during pregnancy (**B**,** C**, and **D**). C gorup = control offsprings without propofol exposure; CD = control offsprings without propofol exposure, with 7,8 DHF injecton 2 hrs before each MWM trail. P = maternal propofol exposed offspring; PD = maternal propofol exposed offspring, with 7,8 DHF injecton 2 hrs before each MWM trail. I group = 20% intralipid exposure offspring. *P group *versus* C group, *P* < 0.05; ^#^ PD group *versus* P group, *P* < 0.05. ^&^ PD group *versus* C group, *P* < 0.05.

Spatial learning and memory of the offspring was tested with MWM analysis on day P30. Preliminary results revealed no obvious gender differences in learning and memory; therefore, the learning and memory results of female and male offspring were combined. Compared to the control group, the offspring in the propofol exposure group required additional time (escape latency) to find the hidden platform but had shorter target travelling time and shorter platform‐crossing times (Fig. 3). Learning and memory in the 20% intralipid group (I group) was not significantly different from that in the control group (Fig. 3B–D). This is consistent with previous reports showing that 20% intralipid infusion (0.4 mg/kg/min.) during late pregnancy had no significant effect on learning and memory, hippocampal neuronal health or synaptophysin levels in rat offspring 52. These results indicate that maternal exposure to propofol during late pregnancy impairs learning and memory in rat offspring.

TrkB agonist 7,8‐DHF binds with high affinity to TrkB receptors and can activate the downstream signalling cascade 53, thereby improving learning and memory. To determine whether impaired learning and memory can be reversed by a TrkB agonist, 5 mg/kg of 7,8‐DHF was intraperitoneally injected in rat offspring 2 hrs before each MWM trial. Selection of the dosage and injection timing was based on previous studies showing that intraperitoneal injection with 5 mg/kg of 7,8‐DHF prevented long‐term spatial memory injury that resulted from restraint stress 54. The results showed that the offspring in the PD group had better learning and memory than those in the P group (Fig. 3). However, the learning and memory in the PD group was still worse than that in the control group (Fig. 3). 7,8‐DHF had no significant effect on learning and memory in rat offspring that were not exposed to propofol during pregnancy (Fig. 3B–D). These results indicate that 7,8‐DHF can significantly improve the impaired learning and memory induced by propofol but cannot reverse these impairments completely.

### Decreased BDNF and TrkB protein levels in the hippocampus of rat offspring and the mitigating effects of DHF treatment

**Figure 4:**
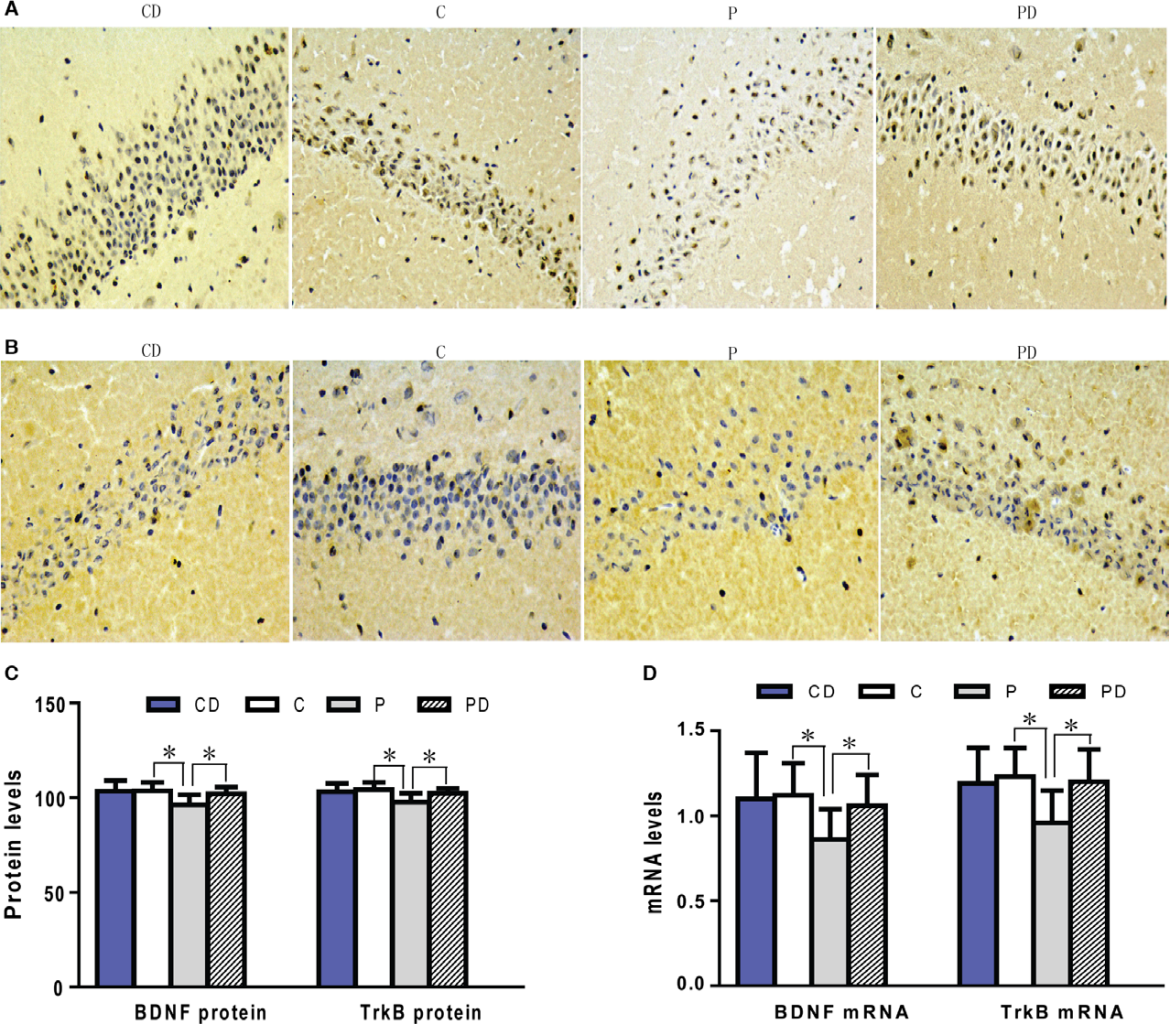
Decreased expression levels of BDNF and TrkB in the offspring rats’ hippocampus and the modulatory effects of DHF treatment. Immunohistochemistry staining revealed that the protein levels of BDNF and TrkB in the propofol exposure group (P group) were significant less than those in the control group (C group) (**A**–**C**). TrkB agonist (7,8‐DHF) reversed the expression of BDNF and TrkB. The protein levels of BDNF and TrkB in the 7,8‐DHF treatment group (PD group) were higher than those in the P group (**A**–**C**). Further, 7,8‐DHF did not change the protein levels of BDNF and TrkB in rat offspring that had not been exposed to propofol (CD group) (**A**–**C**). The changes of BDNF and TrkB mRNA expressions were similar to their proteins (**D**). (**A**) BDNF protein immunohistochemistry stain in offspring's hippocampus (400×); (**B**) TrkB protein immunohistochemistry stain in offspring's hippocampus (400×); (**C**) Data represents mean ± S.E. of 20 offspring rats in each group. (**D**) Real time PCR results of BDNF and TrkB mRNA expressions. Data represents mean ± S.E. of 20 offspring rats in each group, *P < 0.05.

To determine whether BDNF and TrkB are involved in the learning and memory impairments observed following maternal propofol exposure, the protein levels of BDNF and TrkB in hippocampus tissues of the offspring were tested. Because 20% intralipid exposure did not alter learning and memory in the offspring (Fig. 3B–D), we did not examine the protein levels in the 20% intralipid exposure group. Immunohistochemistry staining revealed that BDNF was expressed abundantly both in the cytoplasm and nucleus (most abundant in the nuclear membrane) in the hippocampus; however, TrkB was mainly expressed in the cytoplasm without distribution in the nucleus. The protein levels of BDNF and TrkB in the propofol exposure group (P group) were significant less than those in the control group (Fig. 4), suggesting that the observed impairments in learning and memory may be related to the decreased protein levels of BDNF and TrkB.

To further verify whether the BDNF‐TrkB signalling pathway is involved in learning and memory impairments, a TrkB agonist was injected in rat offspring 2 hrs before each MWM trial. It was discovered that the protein levels of BDNF and TrkB in the PD group were higher than those in the P group but were still lower than those in the control group (Fig. 4). Furthermore, 7,8‐DHF did not change the protein levels of BDNF and TrkB in rat offspring that had not been exposed to propofol (Fig. 4A–C). These results reveal that TrkB agonists can reverse the down‐regulation of TrkB and BDNF proteins caused by propofol exposure but cannot alleviate the noxious effects completely. These results confirm that the learning and memory impairments induced by propofol exposure during late stages of pregnancy correlate with decreased expression of BDNF and TrkB proteins.

### Decreased mRNA levels of BDNF and TrkB in the hippocampus of rat offspring and the modulatory effects of DHF treatment

To explore whether the observed down‐regulation of BDNF and TrkB proteins is caused by inhibition of gene expression, the mRNA levels of BDNF and TrkB was measured by real‐time PCR in hippocampus tissues. We found that the levels of both BDNF and TrkB mRNA were significantly lower in the P group than those in the control group (Fig. 4D) and suggests that down‐regulated mRNA expression causes the observed decrease in protein abundance.

To further verify whether the decreased BDNF and TrkB protein levels are caused by inhibition of gene expression, the mRNA levels of BDNF and TrkB in the PD group were compared to those in the P group and control group. It was discovered that that the mRNA levels of both BDNF and TrkB were significant higher in the PD group than those in the P group but lower than those in the control group (Fig. 4D). 7,8‐DHF had no significant effects on mRNA expression of BDNF or TrkB in the rat offspring that were not exposed to propofol (Fig. 4D). These results further verify that down‐regulated mRNA expression is involved in the decrease of BDNF and TrkB protein levels induced by propofol exposure during late stages of pregnancy.

### Maternal propofol exposure decreases TrkB phosphorylation that is inhibited by DHF treatment

**Figure 5:**
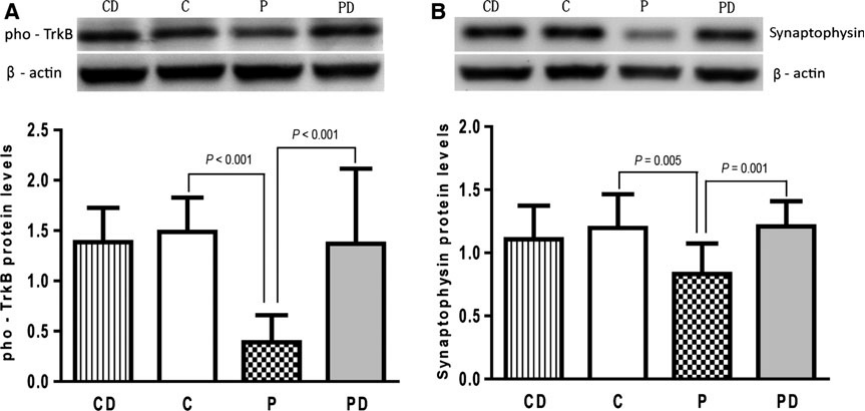
Maternal propofol exposure decreased the phosphorylation of TrkB and the expression of synaptophysin and the reversed effect of DHF treatment. (**A**) Phosphorylated levels of TrkB analysed by western blot. Maternal propofol exposure resulted in decreased levels of phospho‐TrkB protein in offspring rats’ hippocampus. Significantly increased phospho‐TrkB protein levels were observed in the PD group compared to the P group. However, 7,8‐DHF did not affect the phosphorylation of TrkB in offspring rats that without exposed to propofol (CD group). (**B**) Synaptophysin protein expression levels detected by western blot. Maternal exposure to propofol decreased the expression of synaptophysin, a synaptic protein marker in offspring hippocampus. DHF treatment reversed the down ‐ regulated expression of synaptophysin. The offpring rats in propofol exposed group had lower synaptophysin levels than control group (*P* = 0.005) and DHF treated group (PD group) (*P* = 0.001). DHF had no effect on the expression of synaptophysin in offspring rats without exposed to propofol during late pregnancy (CD group). Data represents mean ± S.D. of 10 male and 10 female offspring rats in each group.

Tyrosine kinase B is autophosphorylated after binding to its ligand and the phosphorylation of TrkB facilitates long‐lasting synapse formation 55; therefore, we investigated the phosphorylation of TrkB in the current study. We found that maternal propofol exposure resulted in decreased levels of phospho‐TrkB protein. Significantly increased phospho‐TrkB protein levels were observed in the PD group compared to the P group (Fig. 5A), suggesting that 7,8‐DHF prevented the phosphorylation of TrkB. However, 7,8‐DHF did not affect the phosphorylation of TrkB in rat offspring from the CD group (Fig. 5A). These results indicate that maternal exposure to propofol inactivates the BDNF‐TrkB signalling pathway that can be reactivated by treatment with DHF.

### Maternal propofol exposure down‐regulates synaptophysin expression that is restored by DHF treatment

Synaptophysin hippocampal protein levels were detected by western blot. It was discovered that maternal propofol exposure during late pregnancy markedly down‐regulated synaptophysin expression in the hippocampus; however, this synaptophysin down‐regulation was significantly reduced in animals treated with DHF. Synaptophysin protein levels in the propofol exposed group were significantly lower than those in the control group and DHF‐treated group. Significant differences in synaptophysin levels were not observed when comparing between the C and CD groups (Fig. 5B). Synaptophysin is a synaptic protein marker and plays a critical role in memory formation 56. Therefore, these results suggest that maternal propofol exposure decreases synaptic plasticity.

## Discussion

The results in present study showed that maternal exposure to propofol on day E18 impair learning and memory of rat offspring, inhibit expression of BDNF, TrkB and synaptophysin, and inhibit phosphorylation of TrkB in the hippocampus. Tyrosine kinase B agonist 7,8‐DHF significantly improved learning and memory, blocked down‐regulation of BDNF, TrkB and synaptophysin and facilitated phosphorylation of TrkB. Therefore, these results indicate that maternal exposure to propofol during late stages of pregnancy impair learning and memory by inactivating the BDNF‐TrkB signalling pathway in the hippocampus of offspring, and TrkB agonists may ameliorate these effects.

The body temperature, heart rate, respiratory rate, blood pressure and SPO_2_ of the maternal rats were monitored during propofol infusion. No significant changes were observed for any of these indicators; however, two maternal rats died during anaesthesia induction and 1 died during propofol infusion. The levels of various artery blood gases were analysed at the end of propofol infusion. The blood gas levels of maternal rats in the propofol exposure group were not significantly different from those in the control group (Table 1). Therefore, learning and memory impairments of the offspring were not caused by pathological disorders induced by maternal propofol exposure. Propofol did not affect physical development, and there were no significant differences in bodyweight or dyskinesia between propofol exposure and control groups. In late‐pre‐term lambs subjected to asphyxia, it has been shown that maternal propofol anaesthesia results in less cardiac injury than does isoflurane anaesthesia 49, and cardiac protection from prenatal asphyxia has also been observed 49, 57. This suggests that the learning and memory impairments observed in the current study were induced by maternal propofol exposure instead of physical differences. Day P30 in rats is equivalent to human adolescence. Therefore, the results herein indicate that maternal propofol exposure can impair learning and memory in mature rat offspring. These results are similar to previous reports in which a higher dose of propofol was used with shorter infusion times on day E18 that led to brain damage and permanent learning and memory dysfunction in offspring 52, 58. Compared to isoflurane, treatment of the ovine maternal‐foetal unit with propofol has been shown to improve EEG results in preterm lambs suffering from severe asphyxia 50. However, our study has considered the long‐term outcome of maternal propofol exposure and its effects on learning and memory in offspring. Propofol has been widely used in pregnant woman for sedation 32, 33, cesarean delivery 34, 35, 36 and nonobstetric surgery 37, 38. Clinical data indicates that nonobstetric surgeries during pregnancy are safe for mothers and their foetuses 40, 41, 42, 43, 44, 45, 46, 47, 48. However, most of these studies have been focused on the physical development of the foetus without examining the long‐term effects on learning and memory throughout later developmental stages.

Studies have indicated that propofol may impair learning and memory by preventing synaptic plasticity, especially LTP, and facilitates the conception and maintenance of LTD in the CA1 areas of the hippocampus 59. Previous studies have shown that learning and memory impairments induced by propofol involve inhibition of NMDA and AMPA receptors 60. Some studies have indicated that propofol may diminish learning and memory by inhibiting nicotinic acetylcholine 61 and 5‐HT receptors 62. Together, these findings suggest that propofol can impair learning and memory by affecting the expression of multiple genes and by altering proteins interactions. Brain derived neurotrophic factor was first studied by German neurobiologist Barde in 1982 63. Brain‐derived neurotrophic factor is mainly synthesized by the brain and is distributed throughout the CNS, especially the hippocampus and cerebral cortex. Brain‐derived neurotrophic factor plays an important role in the growth, development, differentiation and repair of neurons after injury. Brain‐derived neurotrophic factor promotes Trk dimerization and autophosphorylation by binding to the TrkB receptor and activating intracellular signal transduction pathways. Brain‐derived neurotrophic factor also plays a critical role in regulating the plasticity and function of synapses *via* TrkB receptor activation 64, 65. Tyrosine kinase B deficient TrkB‐CRE transgenic mice 66 show complete MWM test failure and also exhibit poor performance in the eight‐arm radial maze task (only exhibiting the passive avoidance response). This suggests that the BDNF‐TrkB signalling pathway plays an important role in complex learning and memory phenomena. Herein, the hippocampus tissue levels of BDNF and TrkB mRNA transcripts and proteins were detected with real‐time PCR and IHC, respectively. It was discovered that the offspring of propofol exposure group had significantly lower levels of BDNF and TrkB than did the control group. The phosphorylated TrkB (phospho‐TrkB) levels and synaptophysin protein levels in the hippocampus were also reduced in the propofol exposure group. Phosphorylation of TrkB is critical to memory formation. Tyrosine kinase B phosphorylation‐deficiency impairs spatial memory and compromises hippocampal LTP 67. The BDNF‐TrkB signalling pathway facilitates synaptic plasticity. Synaptophysin is a synaptic protein marker that plays a critical role in memory formation 56. Our western blot results reveal that the levels of synaptophysin in the propofol exposure group are significantly lower than those in the control group (Fig. 5B). Therefore, our results indicate that the learning and memory impairments induced by maternal propofol exposure correlate with inhibition of BDNF and TrkB expression and inhibition of TrkB phosphorylation, which suggests that the BDNF‐TrkB signalling pathway is involved in the learning and memory impairments caused by maternal propofol exposure. Previous studies have shown that adult rat exposed to propofol can also lead to spatial learning and memory impairments and inhibition of BDNF expression in the hippocampus 68.

To verify the role of the BDNF‐TrkB signalling pathway in learning and memory, the TrkB agonist 7,8‐DHF was used to treat rat offspring. 7,8‐DHF is a recently discovered selective agonist of the TrkB receptor. 7,8‐DHF is a flavonoid compound that displays neuroprotective effects in neurological disorders such as Alzheimer disease 69 and stroke 53. 7,8‐DHF is the first medicine that can mimic the effects of BDNF. It can activate the TrkB receptor in the brain, thereby improving learning and memory, repairing neuronal injury in neurodegenerative disease and prohibiting neuronal apoptosis. In the present study, we have shown that 7,8‐DHF can improve propofol‐induced learning and memory impairments and can up‐regulate the decreased levels of BDNF and TrkB transcripts and proteins in the hippocampus of rat offspring that are exposed to propofol. Moreover, 7,8‐DHF can also potentiate the phosphorylation of TrkB (Fig. 5A). However, DHF had no effect on BDNF and TrkB expression or TrkB phosphorylation in offspring from the CD group (Figs 4 and 5A). These results further implicate a critical role for the BDNF‐TrkB signalling pathway in the learning and memory impairments induced by maternal propofol exposure.

Synaptophysin is a synaptic protein marker and provides a structural basis for synaptic plasticity 56. Others have shown that neuronal plasticity markers, such as synaptophysin, growth associated protein 43 (GAP43) and post‐synaptic density protein 95 (PSD95), are dependent on BDNF processing and subsequent TrkB signalling pathways 22. Our results showed that maternal propofol exposure decreased the protein levels of synaptophysin in the hippocampus of rat offspring (Fig. 5B). These findings are consistent with a previous report showing that propofol exposure on day E18 increased cleaved caspsase‐3 levels, decreased neuronal density, reduced synaptophysin levels and caused persistent learning deficits in the rat offspring 52. Herein, treatment with DHF was shown to up‐regulate decreased synaptophysin levels and was discovered to improved learning and memory impairments (Fig. 5B). However, DHF had no such effects on rat offspring that were without prenatal propofol exposure (Fig. 5B). These results further confirm that the BDNF‐TrkB signalling pathway is involved in the learning and memory impairments that are caused by maternal propofol exposure during late pregnancy stages.

The current results show that 7,8‐DHF can improve learning and memory impairments in offspring exposed to propofol, but the impairments were not completely eliminated. The possible reasons for the incomplete rescue are that first, the dosage of 7,8‐DHF may not be sufficient, although 5 m/kg of 7,8‐DHF has been shown to reverse memory impairments caused by immobilization stress 54. Second, the treatment time may have been insufficient. We only treated the offspring 2 hrs before each MWM trial, but perhaps several consecutive weeks of 7,8‐DHF injection are needed. Finally, the timing of the 7,8‐DHF injections may have been ineffective. Rat brain development is at its maximum from days E16 to P7; therefore, treatment with 7,8‐DHF either immediately following propofol exposure or soon after pup delivery might be more effective. Additional investigations are required to resolve the aforementioned issues.

Brain‐derived neurotrophic factor and TrkB can facilitate the dimerization and phosphorylation of TrkB and can thereby activate MAPK, PIK3 and phospholipase C‐γ1 signalling pathways to protect hippocampal neurons from damage induced by glutamate 70 and ischaemia 71. However, because of the short half‐life of BDNF, studies on the clinical effects of BDNF have not been informative 53. The neuroprotective effects of 7,8‐DHF have been observed in rodent models of neurological diseases, including stress 54, stroke 71, depression 72, ageing 73 and Alzheimer disease 74. In addition to potentiating the expression of TrkB, 7,8‐DHF can improve learning and memory by inducing synaptic expression of the AMPA GluA1 receptor 75 and increasing dendritic spine density 76. This is the reason why we selected a TrkB agonist other than BDNF to reverse the noxious effects of propofol exposure herein.

In the current study, we use the MWM to evaluate learning and memory. To provide a more comprehensive assessment of learning and memory effects in future studies, multiple behavioural test systems should be used, such as the Barnes maze test, the eight‐arm radial maze task and the fear conditioning test.

In conclusion, the current results indicate that maternal propofol exposure during late pregnancy stages impairs spatial learning and memory in rat offspring by disturbing the BDNF‐TrkB signalling pathway. Additionally, the TrkB agonist 7,8‐DHF can ameliorate learning and memory impairments by facilitating the expression of BDNF and TrkB.

## Conflicts of interest

The authors confirm that there are no conflicts of interest.

## Author contribution

Conceived and designed the experiments: Foquan Luo, Weilu Zhao. Performed the experiments: Liang Zhong, Liuqin Wu, Yunlin Feng, Jiamei Lin, Shengqiang Wang, Tianyin Liu, Xuexxue You, Wei Zhang. Analysed the data: Foquan Luo, Weilu Zhao. Contributed reagents/materials/analysis tools: Liang Zhong. Wrote the article: Foquan Luo, Yunlin Feng, Liang Zhong.
